# Reactive Oxygen Species as Potential Drivers of the Seed Aging Process

**DOI:** 10.3390/plants8060174

**Published:** 2019-06-14

**Authors:** Katarzyna Kurek, Beata Plitta-Michalak, Ewelina Ratajczak

**Affiliations:** 1 Institute of Dendrology, Polish Academy of Sciences, Parkowa 5, 62-035 Kórnik, Poland; katarzynakurek95@gmail.com; 2 Department of Biology, Technische Universität Darmstadt, D64287 Darmstadt, Germany

**Keywords:** aging seeds, reactive oxygen species (ROS), antioxidant system, DNA damage, methylation

## Abstract

Seeds are an important life cycle stage because they guarantee plant survival in unfavorable environmental conditions and the transfer of genetic information from parents to offspring. However, similar to every organ, seeds undergo aging processes that limit their viability and ultimately cause the loss of their basic property, i.e., the ability to germinate. Seed aging is a vital economic and scientific issue that is related to seed resistance to an array of factors, both internal (genetic, structural, and physiological) and external (mainly storage conditions: temperature and humidity). Reactive oxygen species (ROS) are believed to initiate seed aging via the degradation of cell membrane phospholipids and the structural and functional deterioration of proteins and genetic material. Researchers investigating seed aging claim that the effective protection of genetic resources requires an understanding of the reasons for senescence of seeds with variable sensitivity to drying and long-term storage. Genomic integrity considerably affects seed viability and vigor. The deterioration of nucleic acids inhibits transcription and translation and exacerbates reductions in the activity of antioxidant system enzymes. All of these factors significantly limit seed viability.

## 1. Seed Classification

**Figure 1:**
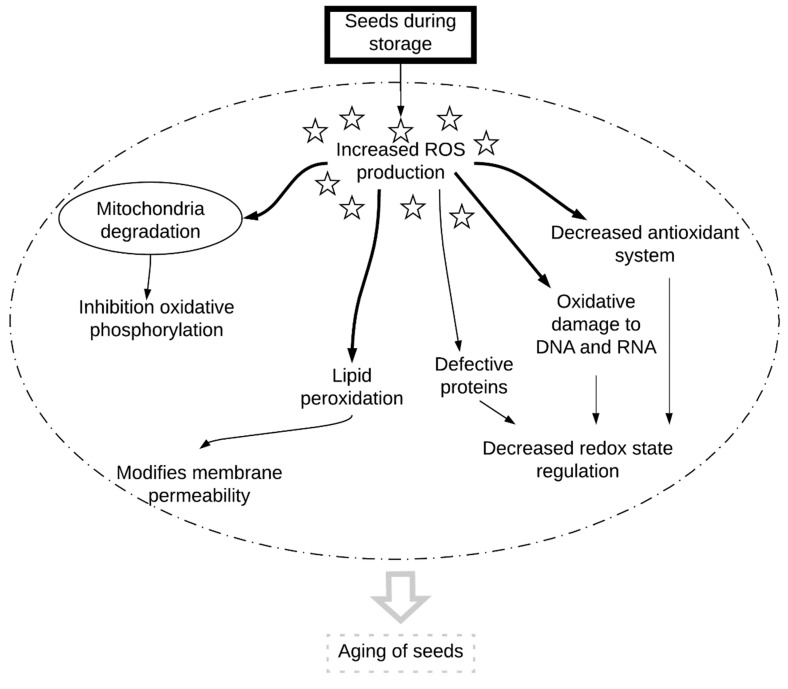
The role of reactive oxygen species (ROS) in seed aging.

During storage, seeds are exposed to oxidative damage caused by reactive oxygen species (ROS). Oxidative damage results in lower germination of seeds and the loss of their viability [1,2]. The role of ROS as the main factor generating the process of seed aging is still being elucidated (Figure 1). 

The rate of damage during storage depends on the properties of seeds. Seeds are divided into three categories: orthodox, recalcitrant, and intermediate. First, orthodox seeds tolerate drying up to 0.03–0.07 g^−1^ H_2_O DW (dry weight) [3,4] and storage at approximately −10 °C. Orthodox seeds become tolerant to drying during maturation [5,6]. Proteome, transcript, and gene analyses have provided some data on orthodox seed tolerance to desiccation. This tolerance is due to limited metabolism (following drying) and an extensive antioxidant system, the activity of which depends on a complex signaling network [7,8,9,10]. The desiccation tolerance of orthodox seeds also relies on an appropriate content of soluble sugars content, mainly the raffinose family oligosaccharides (RFOs) [11,12] and the presence of protective late embryogenesis abundant (LEA) proteins [13,14,15,16] and heat shock proteins (HSPs) [10,17]. Furthermore, the lifespan of orthodox seeds can be extended when they enter the vitreous state [3,18], with many metabolic processes reduced to a minimum or even stopped completely [19]. Orthodox seeds are produced by crops (i.e., *Triticum aestivum* L. and *Zea mays* L.), herbaceous plants (i.e., *Arabidopsis thaliana* L. and *Medicago truncatula* L.), and trees (i.e., *Acer platanoides* L. and *Fraxinus excelsior* L.) (https://data.kew.org/sid/).

Recalcitrant seeds do not tolerate long-term storage and severe drying, with a limit of 0.2–0.3 g H_2_O g^−1^ DW. The third category comprises intermediate seeds. Roberts [20] classified these seeds as being sensitive to water loss and/or storage at low temperatures. They are, therefore, difficult to preserve using traditional seed banks methods. Recalcitrant and intermediate seeds are produced by species such as *Quercus robur* L. and *Fagus sylvatica* L. in temperate zones and *Persea americana* Mill. and *Coffea arabica* L. in the tropics, respectively (https://data.kew.org/sid/).

The reason for viability loss in recalcitrant seeds *Avicennia*, *Acer pseudoplatanus* L. [9,21,22] and intermediate seeds of *Fagus sylvatica* L. [2] during drying and storage is the accumulation of ROS. ROS accumulation occurs due to the low activity of the antioxidant system and differences in the content of LEA defense proteins content [7]. The sensitivity of these seeds is likely further increased by their structure, which does not protect against mechanical damage [22].

## 2. Reactive Oxygen Species and the Antioxidant System

The production and accumulation of ROS depend on the metabolic and physiological state of the seeds [1,23]. In hydrated seeds, the main sites of ROS production are mitochondria, glyoxysomes, and plasma membrane NADPH oxidases [1]. In dry seeds, ROS are synthesized in a nonenzymatic reaction [24]. They originate as a result of the partial reduction of oxygen, which results in the formation of superoxide anion (O_2_^−•^), hydrogen peroxide (H_2_O_2_), and finally hydroxyl radical ^(•^OH). It is assumed that an imbalance in the intracellular ROS status drives the processes leading to the loss of seed viability [2,25,26]. In accordance with the “free radical theory of aging” [27], the seed viability loss during senescence is caused by excessive production of ROS (O_2_^−•^, H_2_O_2_ and ^•^OH) [2] combined with a reduced antioxidant potential in cells [28] and the gradually accumulated oxidative damage of seed cells. These assumptions have proven true in numerous studies on aging seeds [2,29,30,31,32,33]. ROS oxidize lipids and inactivate enzymes damage the structure and function of proteins and carbohydrates, and modify or disrupt DNA structure [34]. It has been suggested that lipid peroxidation may be the main process associated with the aging of seeds. Lipid peroxidation modifies membrane permeability, which affects the decrease in seed viability [2]

Large changes during the aging of seeds occur in mitochondria because they are the main site of ROS production, so they are more quickly and strongly exposed to oxidative damage than other organelles [35,36,37,38]. During the natural aging of beech (*Fagus sylvatica* L.) seeds, a several-fold increase in H_2_O_2_ was observed in the mitochondria of embryonic axes and cotyledons [2]. Oxidative phosphorylation in mitochondria is one of the main sources of ROS in cells, so the free radical theory of aging may essentially be a mitochondrial theory of aging for plant seeds (Figure 1) [38]. The accumulation of ROS in mitochondria causes dysfunction of their membranes as well as the oxidative damage of mitochondrial proteins and DNA [39], and the consequence of these changes is the inhibition of phosphorylation [40]. The increase in ROS production and accumulation in the mitochondria of aging seeds reduces the activity of the antioxidant system. Other researchers observed a decrease in the activity of the ascorbic-glutathione cycle [35] and the activity of enzymatic antioxidants [36,37].

The activity of ROS and area of ROS molecular action in plant cells are highly dependent on the ability of ROS to move across cell membranes. Hydrogen peroxide, the most stable ROS, easily migrates through membranes over relatively long distances, even in dry seeds, thus contributing to seed aging [2,28,35,41,42,43]. The levels of O_2_^−•^, H_2_O_2_, and ^•^OH increased in the embryonic axes and the cotyledons when beech seeds were stored. A strong negative correlation was observed between germination and the levels of O_2_^−•^ *(r* = −0.9386, *p* = 0.005647 in the embryonic axes, *r* = −0.8411, *p* = 0.035912 in the cotyledons), H_2_O_2_ (*r* = −0.9471, *p* = 0.004139 in the embryonic axes, *r* = −0.9557, *p* = 0.0029 in the cotyledons) and ^•^OH (*r* = −0.8306, *p* = 0.040428 in the embryonic axes, *r* = −0.955, *p* = 0.002992 in the cotyledons). The level of H_2_O_2_ was most strongly correlated with reduced germination [2]. During aging, the H_2_O_2_ level and lipid peroxidation in cotton seeds increased in relation to reduced germination [44]. Kibinza et al. (2006) [45] showed a linear relationship between the H_2_O_2_ content and germination of sunflower seeds during aging. The loss of viability in wheat seeds during aging was dependent on H_2_O_2_ production and the lipid peroxidation level [42].

However, it is important to keep in mind that low levels of ROS seem to be favorable for plant cells, as ROS serve as signaling particles, initiating a number of molecular, biochemical, and physiological processes [2,46,47]. ROS signaling is required for seed dormancy breaking and the stimulation of germination [1,47,48,49,50,51,52,53], probably via the activation of gibberellic acid (GA) synthesis [50,51] and mobilization (oxidation) of storage proteins [49]. Additionally, H_2_O_2_ is regarded as a signal molecule, which participates in the regulation of seed dormancy and germination [54]. A balanced H_2_O_2_ level is beneficial, as it promotes germination, whereas excessive H_2_O_2_ content induces oxidative damage, which prevents or delays germination. According to the “oxidative window” hypothesis, both lower and higher levels of ROS have a negative effect on seed germination, and a positive effect is only possible within a critical range of concentrations [48]. Taken together, these results demonstrate that maintaining redox balance in seed cells considerably affects seed viability during drying [2].

By maintaining ROS balance and cellular homeostasis, the system of antioxidants plays an important role in redox regulation by ROS removal and counteracts potential molecular damage [55,56]. The system involves antioxidant enzymes, such as guaiacol peroxidase (POX), catalases (CATs), and superoxide dismutases (SODs) and enzymes of the ascorbate-glutathione cycle, such as ascorbate peroxidase (APX), dehydroascorbate reductase (DHR), and glutathione reductase (RG), in association with low-molecular-weight antioxidants, e.g., ascorbic acid and glutathione, both reduced (GSH) and oxidized (GSSG). Aging seeds are subject to changes in their antioxidant system (demonstrated mainly using spectrometric methods). In aging cotton seeds, the activity of scavenging enzymes, e.g., peroxidase (POD), CAT, APX, and SOD, decreased [44]. Similarly, SOD, CAT, and RG activity declined in sunflower seeds during accelerated aging [57]. A similar decrease in enzymatic activity was demonstrated in wheat during accelerated aging [11]. Kibinza et al. [45] showed that the decreasing activity of SOD, CAT, and GR was related to progressive water content decline. These authors have also demonstrated a relationship between enzymatic activity and seed viability, and the activity of the enzymes was higher during seed germination. The sunflower seeds were subjected to the aging process [28]. Seeds were equilibrated for 24 h at 20 °C, in closed flasks with water to obtain seeds with a water content of 0.29 g H_2_O/g DM (in relation to dry matter), and placed at 35 °C for different periods (3, 5, 7, and 9 days of aging). The aging process caused a decrease in CAT activity, CAT protein content, and CAT transcript accumulation. The decreased CAT protein content (assessed by immunoblot after SDS-PAGE) is probably due to induced oxidation during aging. In our opinion, the reduced CAT transcript accumulation (assessed by northern blot) may result from oxidative stress, which may be indicative of ROS accumulation and temperature effect. In beech seeds undergoing natural aging, the decrease in ascorbic acid (ASA) content in seeds stored for 2 years was twice as high as that in 5-year-old seeds, and the level of GSSG was higher than that of GSH, which indicated the occurrence of oxidative stress in the seeds. A positive correlation was observed between the germination capacity of seeds and the ASA and GSH contents [21]. Ratajczak et al. [2] reported decreased activity of CAT in beech seeds and a strong negative correlation between the level of H_2_O_2_ and CAT activity (*r* = −0.9177 in embryonic axes and *r* = −0.9217 in cotyledons). In aging pea seeds, the activity of glucose-6-phosphate (G6PDH) did not change during aging, while the activity of RG was lower in aging seeds. In pea seeds undergoing aging, Chen et al. [39] observed increased levels of GSSG, an increase towards a more oxidizing half-cell reduction potential E_GSSG/2GSH_ value and decreased germination.

ROS may modify cellular redox potential [46,54] by altering the redox state of GSH/GSSG [48]. Changes in the E_GSSG/2GSH_ value have been identified as an effective marker of oxidative stress [58] and unfavorable changes in seed viability and vigor [59].

Redox regulation, including the presence of peroxiredoxins (Prxs) in maturing seeds, may significantly affect seed viability during long-term storage (Ratajczak, unpublished data). Prxs are thiol-specific antioxidant proteins that catalyze the detoxification of alkyl hydroxides, nitrogen peroxides, and especially hydrogen peroxide [60]. Prxs performs three important functions: (1) antioxidation; (2) control of the redox state of plant cell development and adaptation to environmental conditions; and (3) modulation of cell signaling [60]. The activity of Prxs is primarily regulated by the action of thiol proteins, such as glutaredoxin and thioredoxin [60,61]. The combination of these factors may improve seed resistance to oxidative stress and diminish cell damage [54]. Thus, the redox state influences gene expression, leading to determining, in conclusion, the disruption and breakdown of nucleic acids and changes in many structural and nuclear proteins [39].

During desiccation and imbibition of *A. thaliana,* seeds upregulation of the *At*OGG enzyme that acts as apurinic/apyrimidinic DNA glycosylase/lyase and removes oxidatively damaged guanosines from DNA observed [62]. This observation was consistent with results showing a significant decrease in oxidized nucleobases in transformed *Arabidopsis* protoplasts overexpressing *At*OGG [62]. Therefore, this enzyme contributes to seed resistance to detrimental ROS activity by decreasing the number of their products and finally increasing the viability of seeds in controlled aging conditions. Consequently, in sum, a combination of multiple factors including ROS scavengers, enzymatic antioxidants, and other enzymes removing ROS-induced damage (e.g., those associated with the base excision repair pathway) improve seed resistance to oxidative stress and diminish cell damage [54,62]. 

## 3. Disruption of Genetic Material

Although the level of DNA damage in seeds can vary greatly depending on seed structure and climatic conditions [63,64], DNA damage in stored seeds is thought to mainly originate from ROS-induced oxidative stress. The DNA nucleotide damage by ROS is caused by the oxidation of sugar residues and strand rupture, as both deoxyriboses and nucleobases are susceptible to ROS oxidative damage [65]. The most aggressive ROS that causes DNA fragmentation is ^•^OH [2]. ROS disrupt deoxyribose in DNA molecules mainly by releasing a hydrogen atom [34]. Degradation via oxidation cleaves carbohydrate-phosphate structures, causing single- and double-strand breaks, the chemical modification of bases and the cross-linking of carbonyl and amine groups [66]. The oxidation of nucleic acids occurs mainly in seeds with low water content (approximately 4%) [39]. ROS induces approximately 20 different types of DNA nucleotide damage, the most common of which is the modification of guanine (G), resulting in the formation of 8-oxoguanine (8-oxoG), which is considered a biomarker of oxidative stress. The presence of this base in DNA contributes to transversion mutations (GC→TA) [67]. The level of 8-oxoG increases during aging [68]. Importantly, most studies on 8-oxoG have examined animal tissues, and this type of guanine modification is poorly understood in plants [67]. However, it was shown that imbibition of dried *A. thaliana* seeds resulted in a significant increase in 8-oxoG [62]. Seeds of *Medicago truncatula* and *Shorea robusta* can be subjected to DNA damage due to increased oxidation and fragmentation of DNA, associated with ROS accumulation, over in the course of senescence [69]. Similar disturbances of genetic integrity and DNA fragmentation have also been reported in aging tissues of *Ulmus pumila* L. [31] and in seeds of *Acer platanoides* L. (Plitta-Michalak unpublished data) and *A. thaliana* [64].

DNA damage is harmful to cells, and when this damage is not repaired, it may disturb genome integrity and cause mutations of single or multiple nucleotides. Effective germination of stored seeds requires the DNA damage repair system at the beginning of imbibition [70]. Indeed, seed aging increases the chromosome aberration rate [70,71,72]. Therefore, functional repair mechanisms are crucial for sustaining seed germination and longevity [64,73,74,75]. Waterworth et al. [75] showed that DNA Ligase VI, a plant-specific enzyme that participates in the repair of DNA strand breaks, is crucial for maintaining *A. thaliana* seed viability as *atlig6* mutants displayed significant hypersensitivity to controlled seed aging. Another study by [76] suggested that poly(ADP)polymerase 3 (PARP3) is required for maintaining of *A. thaliana* seed viability during storage. Homologs of this enzyme in mammalian cells catalyze the transfer of ADP-ribose moieties onto proteins related to DNA base repair, thereby recruiting them to damage sites. AtPARP mutants were characterized by delayed germination and lower tolerance to unfavorable storage conditions.

mRNA accumulates in seeds in large amounts, e.g., in *Arabidopsis* seeds deposited mRNA represent more than half of all genes [77]. mRNA in dry seeds is stored until germination [78]. In hydrated seeds, mRNA is linked to posttranscriptional regulation and targeted catalysis of damaged RNA [79]. RNA is more easily damaged than DNA [80]. Degradation of undamaged mRNA is initiated by deadenylation and followed by decapping and 5′–3′ exonuclease degradation or by 3′–5′ exosomal degradation [79]. In aging seeds, both DNA and RNA fragmentation can be used to determine the level of damage [81,82]. Damage to mRNA and rRNA compromises germination progress in seeds [83]. The integrity of total RNA is measured using the RNA integrity number (RIN), which in dry seeds shows that the integrity of total RNA is strong and positively correlated with the germination of seeds [80,82]. Fleming et al. [82] showed that mRNA degradation might occur as a result of nonenzymatic fragmentation. Their assumptions were based on the fact that genome oxidation was not observed and on quantitative polymerase chain reaction (qPCR) showing no blockage of oligo (dT)-primer cDNA synthesis. According to [82], mRNA damage is the result of random fragmentation, which according to the cited authors, is consistent with the hypothesis that mortality arises from oxidation events [84]. Fragmented mRNA can slow down translation and cause the dysfunction of proteins and loss of germination capacity of seeds during storage [29,80,85].

## 4. Changes in DNA Methylation

The premise that genome-wide changes in gene expression modulate plant physiology and development in response to external conditions is the basis for conducting epigenetic studies [86]. Regulatory mechanisms that control the expression of genomic content include epigenetic processes affecting chromatin structure. Enzymatically controlled methylation of cytosines and posttranscriptional modifications of histones, together with histone variants and chromatin conformation regulating factors play key roles in developmental complexity, phenotypic diversity, and the adaptative capacity of the plant [87]. The most extensively characterized modification that shapes the epigenetic landscape is methylation of the carbon at the fifth position (C5) of cytosine. While it does not change the information written in the primary DNA sequence, 5-methylcytosine (m^5^C) regulates genome structure and function by affecting the ability of the molecular machinery to access DNA [86,87,88].

The level of 5-methylcytosine in plants widely ranges from 6% to 30% [89]. In seeds of woody plants, such as *Acer platanoides* L., *Acer pseudoplatanus* L., *Pyrus communis* L., and *Quercus robur* L., global methylation levels have been reported to range from ∼13–22% [86,90,91,92,93]. In plants, the DNA methylation to demethylation ratio may be the major epigenetic mechanism that controls genome function. Indeed, C/m^5^C ratio-related epigenetic events associated with reproduction and seed development have been described. They include, for instance, global demethylation of the vegetative cell genome in order to silence transposable elements in both gametes and the embryo, or extensive DNA methylation changes during seed development [87]. Notably, [94] showed that major classes of seed-related genes have the same methylation profile, whether they are active or not. This observation suggests that DNA methylation does not play a significant role in the regulation of the expression of genes important for seed development. However, within all plant genomes, the distribution of m^5^C is nonrandom, as repetitive regions including transposable elements, centromeric repeats, and rDNA sequences are enriched in modified cytosines. Therefore, it is plausible that any substantial deviation in the C/m^5^C ratio resulting from internal or external signals may have the greatest effect, particularly on these genome elements [86,87].

However, even though many genes encoding seed storage proteins, oil biosynthesis enzymes, or transcriptional factors are located among regions of the genome devoid of DNA methylation at any stage of seed development [94], several previous reports on seeds have indicated that DNA methylation plays a role in seed development and viability [91,95,96,97,98,99]. Therefore, a question has been raised: how is DNA methylation considered at a global scale, rather than at a small, arbitrary fraction of it (gene), related to seed aging and its response to severe stress conditions? Michalak et al. [90] addressed this question while studying the relationship between DNA methylation and severe desiccation in orthodox seeds of wild pear (*Pyrus communis* L.). Severe desiccation down to a 2–3% of moisture content (MC) increased the global DNA methylation level measured in entire embryos. A similar tendency was observed when seeds were maintained for up to one year in conditions optimal for short term storage. That observation suggests that an increase in m^5^C level is beneficial to orthodox seeds and that this process, which is thought to affect genome structure, may play a role in their stabilization and protection during extreme water withdrawal and optimal storage that does not affect seed viability. In another study, [86] DNA methylation changes in embryonic axes and cotyledons of orthodox and recalcitrant seeds have been demonstrated. DNA methylation level has decreased gradually in desiccated embryonic axes of Norway maple (*A. platanoides* L.), but no changes in methylation were noticeable in a second embryo tissue: cotyledons. In the same study, however, changes in methylation were recorded in both embryonic axes and cotyledons of sycamore maple (*A. pseudoplatanus* L.), which has *recalcitrant* seeds [86]. Based on this research, some conclusions can be made. First of all, even though both *P. communis* and *A. platanoides* seeds are classified as *orthodox*, they differ in their tolerance to extreme desiccation is visible. Seeds of wild pear are much more resistant to extreme water withdrawal, which makes them “true” orthodox seeds. The difference is noticeable based on the germination results as well as genomic DNA methylation responses to desiccation. This observation supports the claim that seed exhibit a wide spectrum of responses to drying rather than the rigid responses defined by the different categories [8]. Second, changes in global DNA methylation level induced by desiccation seem to be seed category- and tissue-specific. These changes are a plausible reflection of different physiological responses of orthodox and recalcitrant cotyledons to water deficit. The higher sensitivity to desiccation of cotyledons derived from recalcitrant seeds was previously shown based on increased permeability of plasma membrane and electrolyte leakage as well as disruption in the electron transport chain [86,100,101].

The problem of seed aging was also addressed in several investigations. Michalak et al. [91] showed that aging-related changes in genomic m^5^C levels in recalcitrant *Q. robur* seeds were not linear but rather associated with a significant drop in viability. A possible explanation for these changes is that after 12 months of storage, *Q. robur* seeds started to deteriorate because their mechanisms of antioxidative defense began to be insufficient. Epigenetic regulation is complex, and it is only when accumulations of changes, for instance, in m^5^C levels, influence seed viability only when they reach a certain critical point. Further investigation of *A. thaliana* seedlings revealed that plant genomic DNA might undergo demethylation during plant aging due to a reduction in DNA methylation processes and activation of DNA demethylation. Asymmetric hypermethylation of cytosine in 5S ribosomal RNA was correlated with seed aging in *A. thaliana* as well. Older plants displayed reduced expression of chromomethyltransferase 3 (CMT3) and methyltransferase (MET1) and enhanced expression of repressor of silencing 1 (ROS1) demethylase [99]. A decrease in DNA methylation was also visible during the cotyledon senescence of *Gossypium hirsutum* orthodox seeds [102].

However, the question of the processes responsible for DNA demethylation still needs to be addressed, particularly in the case of desiccated seeds. It is known that the initial lowering of the moisture content of seeds does not have a negative impact on cell metabolism, in contrast to dehydration. In seeds in such a state, the C/m5C ratio may be regulated by both enzymatic and ROS-related activities. However, when water withdrawal proceeds and reaches the threshold MC of 26%, at which point only bound water is observed in recalcitrant seeds, active cellular control may no longer be possible, as at such a level of dehydration, achieving the proper conformation and activity of enzymes becomes problematic [86,103]. Nevertheless, it is known that ROS are the main factors reducing the vitality of seeds during storage [2]. ROS might influence DNA methylation as a result of m^5^C oxidation [91,104] and finally, it might contribute to demethylation of cytosines via, e.g., the base excision repair pathway. It was demonstrated, however, in in vitro conditions, that the formation of 5-hydroxymethylcytosine (hm^5^C) and 5-formylcytosine (f^5^C) represents ^•^OH-induced oxidation products of m^5^C [105]. Moreover, ten–eleven translocation methylcytosine dioxygenases (TETs), which catalyze the conversion of m^5^C to hm^5^C, f^5^C, and 5-carboxylcytosine (c^5^C) in mammalian cells are not found in plants [106]. Therefore, the nature of m^5^C oxidation, whether this modification originates from ROS activity, and its role in plant genome demethylation during water deficit conditions still needs to be investigated.

## 5. Conclusions and Perspectives

Seeds exhibit high genetic diversity and are therefore considered the best method of natural protection of genetic material variability, in contrast to somatic tissues [107]. For this reason, seed storage in seed banks is a vital solution for protecting genetic material. However, loss of viability due to seed aging poses a significant challenge to storage. Therefore, we need to learn more about the mechanisms that regulate seed aging in order to explain how aging affects seed genetic material. Doing so may help us determine the quality of regenerated material. Numerous studies have investigated possible biochemical, physiological [33,58,108,109], and genetic markers [31,103] for assessing seed quality (evaluation of the degree of seed aging). The identification of an appropriate marker would make it easier to monitor seed viability during long-term storage [59].

While analyzing the seed aging process, we should bear in mind that this process is a combined result of unfavorable physiological, biochemical, molecular, and metabolic changes occurring in seed cells. This complexity is why it is so difficult to determine why seeds age and to find a marker that can quickly and reliably monitor the degree of seed aging. Ratajczak et al. [38] suggest that mitochondria are the main initiators of the aging process of seeds. Aging considerably affects genome destabilization, and the lack of an active repair system reduces seed viability and vigor. Seed aging has become a global issue, with consequences that are highly unfavorable for the economy, as the loss of seed viability translates into a lack of material for reproduction.

## References

[1] Kumar S.P.J., Prasad S.R., Banerjee R., Thammineni C. (2015). Seed birth to death: Dual functions of reactive oxygen species in seed physiology. Ann. Bot..

[2] Ratajczak E., Małecka A., Bagniewska-Zadworna A., Kalemba E.M. (2015). The production, localization and spreading of reactive oxygen species contributes to the low vitality of long-term stored common beech (*Fagus sylvatica* L.) seeds. J. Plant Physiol..

[3] Walters C., Hill L.M., Wheeler L.J. (2005). Dying while dry: Kinetics and mechanisms of deterioration in desiccated organisms. Integr. Comp. Biol..

[4] Ellis R.H., Hong T.D. (2006). Temperature sensitivity of the low moisture content limit to negative seed longevity moisture content relationships in hermetic storage. Ann. Bot..

[5] Berjak P. (2006). Unifying perspectives of some mechanisms basic to desiccation tolerance across life forms. Seed Sci. Res..

[6] Berjak P., Farrant J.M., Pammenter N.W., Jenks M.A., Wood A.J. (2007). Seed desiccation-tolerance mechanisms. Plant Desiccation Tolerance.

[7] Berjak P., Pammenter N.W. (2013). Implications of the lack of desiccation tolerance in recalcitrant seeds. Front. Plant Sci..

[8] Walters C.H. (2015). Orthodoxy, recalcitrance and in-between: Describing variation in seed storage characteristics using threshold responses to water loss. Planta.

[9] Ratajczak E., Ströher E., Oelze M.L., Kalemba E.M., Pukacka S., Dietz K.J. (2013). The involvement of the mitochondrial peroxiredoxin PrxIIF in defining physiological differences between orthodox and recalcitrant seeds of two *Acer* species. Funct. Plant Biol..

[10] Ratajczak E., Dietz K.J., Kalemba E.M. (2018). The occurrence of peroxiredoxins and changes in redox state in *Acer platanoides* and *Acer pseudoplatanus* during seed development. J. Plant Growth Regul..

[11] Lehner A., Mamadou N., Poels P., Côme D., Bailly C., Corbineau F. (2008). Changes in soluble carbohydrates, lipid peroxidation and antioxidant enzyme activities in the embryo during ageing in wheat grains. J. Cereal Sci..

[12] Pukacka S., Ratajczak E., Kalemba E.M. (2009). Non-reducing sugar levels in beech (*Fagus sylvatica*) seeds as related to withstanding desiccation and storage. J. Plant Physiol..

[13] Kalemba E.M., Pukacka S. (2007). Possible roles of LEA proteins and sHSPs in seed protection: A short review. Biol. Lett..

[14] Van den Dries N., Facchinelli F., Giarola V., Phillips J.R., Bartels D. (2011). Comparative analysis of LEA-like 11-24 gene expression and regulation in related plant species within the *Lindernia aceae* that differ in desiccation tolerance. N. Phytol..

[15] Delahaie J., Hundertmark M., Bove J., Leprince O., Rogniaux H., Buitink J. (2013). LEA polypeptide profiling of recalcitrant and orthodox legume seeds reveals ABI3-regulated LEA protein abundance linked to desiccation tolerance. J. Exp. Bot..

[16] Radwan A., Hara M., Kleinwächter M., Selmar D. (2014). Dehydrin expression in seeds and maturation drying: A paradigm change. Plant Biol..

[17] Kaur H., Petla B.P., Kamble N.U., Singh A., Rao V., Salvi R., Ghosh S., Majee M. (2015). Differentially expressed seed aging responsive heat shock protein OsHSP18.2 implicates in seed vigor, longevity and improves germination and seedling establishment under abiotic stress. Front. Plant Sci..

[18] Buitink J., Leprince O. (2008). Intracellular glasses and seed survival in the dry state. Comptes Rendus Biol..

[19] Kranner I., Minibayeva F.V., Beckett R.P., Seal C.E. (2010). What is stress? Concepts, definitions and applications in seed science. N. Phytol..

[20] Roberts E.H. (1973). Predicting the storage life of seeds. Seed Sci. Technol..

[21] Pukacka S., Ratajczak E. (2007). Age-related biochemical changes during storage of beech (*Fagus sylvatica* L.) seeds. Seed Sci. Res..

[22] Berjak P., Pammenter N.W. (2008). From *Avicennia* to *Zizania*: Seed recalcitrance in perspective. Ann. Bot. Lond..

[23] Foyer C.H., Ruban A.V., Noctor G. (2017). Viewing oxidative stress through the lens of oxidative signalling rather than damage. Biochem. J..

[24] Bewley J.D., Bradford K.J., Hilhorst H.W.M., Nonogaki H. (2013). Seeds: Physiology of development, germination and dormancy. Springer.

[25] Bailly C. (2004). Active oxygen species and antioxidants in seed biology. Seed Sci. Res..

[26] Møller I.M., Jensen P.E., Hansson A. (2007). Oxidative modifications to cellular. Annu. Rev. Plant Biol..

[27] Harman D. (1992). Free radical theory of aging. Mutat. Res..

[28] Kibinza S., Bazin J., Bailly C., Farrant J.M., Corbineau F., El-Maarouf-Bouteau H. (2011). Catalase is a key enzyme in seed recovery from ageing during priming. Plant Sci..

[29] Rajjou L., Lovigny Y., Groot S.P., Belghaz M., Job C., Job D. (2008). Proteome-wide characterization of seed aging in *Arabidopsis*: A comparison between artificial and natural aging protocols. Plant Physiol..

[30] Bellani L.M., Salvini L., Dell’ Aquila A., Scialabba A. (2012). Reactive oxygen species release, vitamin E, fatty acid and phytosterol contents of artificially aged radish (*Raphanus sativus* L.) seeds during germination. Acta Physiol. Plant..

[31] Hu D., Ma G., Wang Q., Yao J., Wang Y., Pritchard H.W., Wang X. (2012). Spatial and temporal nature of reactive oxygen species production and programmed cell death in elm (*Ulmus pumila* L.) seeds during controlled deterioration. Plant Cell Environ..

[32] Parkhey S., Naithani S.C., Keshavkant S. (2012). ROS production and lipid catabolism in desiccating *Shorea robusta* seeds during aging. Plant Physiol. Biochem..

[33] Yao Z., Liu L.W., Gao F., Rampitsch C., Reinecke D.M., Ozga J.A. (2012). Developmental and seed aging mediated regulation of antioxidative genes and differential expression of proteins during pre- and post-germinative phases in pea. J. Plant Physiol..

[34] Sharma N.L., Kuniyal J.C., Singh M. (2012). Reactive Oxygen Species, Oxidative Damage, and Antioxidative Defense Mechanism in Plants under Stressful Conditions. J. Bot..

[35] Xin X., Tian Q., Yin G., Chen X., Zhang J., Ng S., Lu X. (2014). Reduced mitochondrial and ascorbate-glutathione activity after artificial ageing in soybean seed. J. Plant Physiol..

[36] Xia F.S., Wang M.Y., Li M.L., Mao P.S. (2015). Mitochondrial structural and antioxidant system responses to aging in oat (*Avena sativa* L.) seeds with different moisture contents. Plant Physiol. Biochem..

[37] Mao C., Zhu Y., Cheng H., Yan H., Zhao L., Tang J., Ma X., Mao P. (2018). Nitric oxide regulates seedling growth and mitochondrial responses in aged oat seeds. Int. J. Mol. Sci..

[38] Ratajczak E., Małecka A., Ciereszko I., Staszak A.M. (2019). Mitochondria are important determinants of the aging of seeds. Int. J. Mol. Sci..

[39] Chen H., Osuna D., Colville L., Lorenzo O., Graeber K., Dennis E.S., Peacock W.J. (2013). Transcriptome-wide mapping of pea seed ageing reveals a pivotal role for genes related to oxidative stress and programmed cell death. PLoS ONE.

[40] Liberatore K.L., Dukowic-Schulze S., Miller M.E., Chen C., Kianian S.F. (2016). The role of mitochondria in plant development and stress tolerance. Free Radic. Biol. Med..

[41] Kalemba E.M., Suszka J., Ratajczak E. (2015). The role of oxidative stress in determining the level of viability of black poplar (*Populus nigra*) seeds stored at different temperatures. Funct. Plant Biol..

[42] Kong L., Huo H., Mao P. (2015). Antioxidant response and related gene expression in aged oat seed. Front. Plant Sci..

[43] Wojtyla Ł., Kubala S.Z., Garnczarska M. (2016). Different modes of hydrogen peroxide action during seed germination. Front. Plant Sci..

[44] Goel A., Sheoran I.S. (2003). Lipid peroxidation and peroxide-scavenging enzymes in cotton seeds under natural ageing. Biol. Plant..

[45] Kibinza S., Vinel D., Côme D., Bailly C., Corbineau F. (2006). Sunflower seed deterioration as related to moisture content during aging, energy metabolism and active oxygen species scavenging. Physiol. Plant..

[46] Foyer C.H., Noctor G. (2016). Stress-triggered redox signalling: what’s in pROSpect?. Plant Cell Environ..

[47] Gniazdowska A., Krasuska U., Czajkowska K., Bogatek R. (2010). Nitric oxide, hydrogen cyanide and ethylene are required in the control of germination and undisturbed development of young apple seedlings. Plant Growth. Regul..

[48] Bailly C., El-Maarouf-Bouteau H., Corbineau F. (2008). From intracellular signaling networks to cell death: The dual role of reactive oxygen species in seed physiology. Comptes Rendus Biol..

[49] Oracz K., El-Maarouf-Bouteau H., Kranner I., Bogatek R., Corbineau F., Bailly C. (2009). The mechanisms involved in seed dormancy alleviation by hydrogen cyanide unravel the role of reactive oxygen species as key factors of cellular signaling during germination. Plant Physiol..

[50] Liu Y., Ye N., Liu R., Chen M., Zhang J. (2010). H_2_O_2_ mediates the regulation of ABA catabolism and GA biosynthesis in *Arabidopsis* seed dormancy and germination. J. Exp. Bot..

[51] Bahin E., Bailly C., Sotta B., Kranner I., Corbineau F., Leymarie J. (2011). Crosstalk between reactive oxygen species and hormonal signalling pathway regulates grain dormancy in barley. Plant Cell Environ..

[52] Krasuska U., Gniazdowska A. (2012). Nitric oxide and hydrogen cyanide as regulating factors of enzymatic antioxidant system in germinating apple embryos. Acta Physiol. Plant..

[53] Wang Y., Li Y., Xue H., Pritchard H.W., Wang X. (2015). Reactive oxygen species (ROS)-provoked mitochondria-dependent cell death during ageing of elm (*Ulmus pumila* L.) seeds. Plant J..

[54] El-Maarouf-Bouteau H., Bailly C. (2008). Oxidative signaling in seed germination and dormancy. Plant Signal. Behav..

[55] Dietz K.J., Mittler R., Noctor G. (2016). Recent progress in understanding the role of reactive oxygen species in plant cell signaling. Plant Physiol..

[56] Noctor G., Reichheld J.P., Foyer C.H. (2018). ROS-related redox regulation and signaling in plants. Semin. Cell Dev. Biol..

[57] Bailly C., Benamar A., Corbineau F., Côme D. (1996). Changes in superoxide dismutase, catalase and glutathione reductase activities as related to seed deterioration during accelerated aging of sunflower seeds. Physiol. Plant..

[58] Kranner I., Birti S., Anderson K.M., Pritchard H.W. (2006). Glutathione half-cell reduction potential: A universal stress marker and modulator of programmed cell death?. Free Radic. Biol Med..

[59] Nagel M., Kranner I., Neumann K., Rolletschek H., Seal C.E., Colville L., Fernández-Marín B., Börner A. (2015). Genome-wide association mapping and biochemical markers reveal that seed ageing and longevity are intricately affected by genetic background and developmental and environmental conditions in barley. Plant Cell Environ..

[60] Dietz K.J. (2011). Peroxiredoxins in plants and cyanobacteria. Antioxid. Redox Signal..

[61] Sevilla F., Camejo D., Ortiz-Espín A., Calderón A., Lázaro J.J., Jiménez A. (2015). The thioredoxin/peroxiredoxin/sulfiredoxin system: Current overview on its redox function in plants and regulation by reactive oxygen and nitrogen species. J. Exp. Bot..

[62] Chen H., Chu P.Y., Zhou Y., Li Y., Liu J., Ding Y., Tsang E.W., Jiang L., Wu K., Huang S. (2012). Overexpression of AtOGG1, a DNA glycosylase/AP lyase, enhances seed longevity and abiotic stress tolerance in *Arabidopsis*. J. Exp. Bot..

[63] Silva-Flores R., Pérez-Verdín G., Wehenkel C. (2014). Patterns of tree species diversity in relation to climatic factors on the Sierra Madre Occidental, Mexico. PLoS ONE.

[64] Waterworth W.M., Footitt S., Bray C.M., Finch-Savage W.E., West C.E. (2016). DNA damage checkpoint kinase ATM regulates germination and maintains genome stability in seeds. Proc. Natl. Acad. Sci. USA.

[65] Mittler R. (2017). ROS Are Good. Trends Plant Sci..

[66] Walters C.H., Reilley A.A., Reeves P.S., Baszczak J., Richards C.H.M. (2006). The utility of aged seeds in DNA banks. Seed Sci. Res..

[67] Johnston J.W., Pimbley I., Harding K., Benson E.E. (2010). Detection of 8-hydroxy-2-deoxyguanosine as a marker of oxidative damage in DNA and germplasm exposed to cryogenic treatments. CryoLetters.

[68] Boesch P., Weber-Lotfi F., Ibrahim N., Tarasenko V., Cosset A., Paulus F., Lightowlers R.N., Dietrich A. (2011). DNA repair in organelles: Pathways, organization, regulation, relevance in disease and aging. Biochim. Biophys. Acta.

[69] Chandra J., Parkhey S., Keshavkant S. (2018). Ageing-regulated changes in genetic integrity of two recalcitrant seeded species having contrasting longevity. Trees.

[70] Fu Y.B., Ahmed Z., Diederichsen A. (2015). Towards a better monitoring of seed ageing under ex situ seed conservation. Conserv. Physiol..

[71] Dmitrov B. (1998). Chromosome damage induced by seed aging in *Crepis capillaris* L.. Prog. Bot. Res..

[72] Chwedorzewska K.J., Bednarek P.T., Puchalski J. (2002). Studies on changes in specific rye genome regions due to seed aging and regeneration. Cell Mol. Biol. Lett..

[73] Biedermann S., Mooney S., Hellmann H., Clark C.C. (2011). Recognition and Repair Pathways of Damaged DNA in Higher Plants.

[74] Balestrazzi A., Confalonieri M., Macovei A., Carbonera D. (2011). Seed imbibition in *Medicago truncatula* Gaertn.: Expression profiles of DNA repair genes in relation to PEG-mediated stress. J. Plant Physiol..

[75] Waterworth W.M., Bray C.M., West C.E. (2015). The importance of safeguarding genome integrity in germination and seed longevity. J. Exp. Bot..

[76] Rissel D., Losch J., Peiter E. (2014). The nuclear protein Poly(ADP-ribose) polymerase 3 (AtPARP3) is required for seed storability in *Arabidopsis thaliana*. Plant Biol..

[77] Nakabayashi K., Okamoto M., Koshiba T., Kamiya Y., Nambara E. (2005). Genome-wide profiling of stored mRNA in *Arabidopsis thaliana* seed germination: Epigenetic and genetic regulation of transcription in seed. Plant J..

[78] El-Maarouf-Bouteau H., Meimoun P., Job C., Job D., Bailly C. (2013). Role of protein and mRNA oxidation in seed dormancy and germination. Front. Plant Sci..

[79] Zhang X., Guo H. (2017). mRNA decay in plants: Both quantity and quality matter. Curr. Opin. Plant Biol..

[80] Fleming M.B., Richards C.M., Walters C. (2017). Decline in RNA integrity of dry-stored soybean seeds correlates with loss of germination potential. J. Exp. Bot..

[81] Kranner I., Chen H., Pritchard H.W., Pearce S.R., Birtić S. (2011). Inter-nucleosomal DNA fragmentation and loss of RNA integrity during seed ageing. Plant Growth Regul..

[82] Fleming M.B., Patterson E.L., Reeves P.A., Richards C.M., Gaines T.A., Walters C. (2018). Exploring the fate of mRNA in aging seeds: Protection, destruction, or slow decay?. J. Exp. Bot..

[83] Rajjou L., Duval M., Gallardo K., Catusse J., Bally J., Job C., Job D. (2012). Seed germination and vigor. Annu. Rev. Plant Biol..

[84] Kranner I. (2005). A modulating role for antioxidants in desiccation tolerance. Integr. Comp. Biol..

[85] Sano N., Permana H., Kumada R., Shinozaki Y., Tanabata T., Yamada T., Hirasawa T., Kanekatsu M. (2012). Proteomic analysis of embryonic proteins synthesized from long-lived mRNAs during germination of rice seeds. Plant Cell Physiol..

[86] Plitta-Michalak B.P., Naskret-Barciszewska M.Z., Kotlarski S.Z., Tomaszewski D., Tylkowski T., Barciszewski J., Chmielarz P., Michalak M. (2018). Changes in genomic 5-methylcytosine level mirror the response of orthodox (*Acer platanoides* L.) and recalcitrant (*Acer pseudoplatanus* L.) seeds to severe desiccation. Tree Physiol..

[87] Bräutigam K., Cronk Q. (2018). DNA methylation and the evolution of developmental complexity in plants. Front. Plant Sci..

[88] Boyko A., Kovalchuk I. (2008). Epigenetic control of plant stress response. Environ. Mol. Mutagen..

[89] Chen T., Li E. (2004). Structure and function of eukaryotic DNA methyltransferases. Curr. Top. Dev. Biol..

[90] Michalak M., Barciszewska M.Z., Barciszewski J., Plitta B.P., Chmielarz P. (2013). Global changes in DNA methylation in seeds and seedlings of *Pyrus communis* after seed desiccation and storage. PLoS ONE.

[91] Michalak M., Plitta-Michalak B.P., Naskręt-Barciszewska M., Barciszewski J., Bujarska-Borkowska B., Chmielarz P. (2015). Global 5-methylcytosine alterations in DNA during ageing of *Quercus robur* seeds. Ann. Bot..

[92] Plitta B.P., Michalak M., Naskręt-Barciszewska M.Z., Barciszewski J., Chmielarz P. (2014). DNA methylation of *Quercus robur* L. plumules following cryo-pretreatment and cryopreservation. Plant Cell Tissue Organ Cult..

[93] Plitta B.P., Michalak M., Bujarska-Borkowska B., Barciszewska M.Z., Barciszewski J., Chmielarz P. (2014). Effect of desiccation on the dynamics of genome-wide DNA methylation in orthodox seeds of *Acer platanoides* L.. Plant Physiol. Biochem..

[94] Lin J.Y., Le B.H., Chen M., Henry K.F., Hur J., Hsieh T.-F., Chen P.Y., Pelletier J.M., Pellegrini M., Fischer R.L. (2017). Similarity between soybean and *Arabidopsi*s seed methylomes and loss of non-CG methylation does not affect seed development. Proc. Natl. Acad. Sci. USA.

[95] Adams S., Vinkenoog R., Spielman M., Dickinson H.G., Scott R.J. (2000). Parent-of-origin effects on seed development in *Arabidopsis thaliana* require DNA methylation. Development.

[96] Xiao W., Brown R.C., Lemmon B.E., Harada J.J., Goldberg R.B., Fischer R.L. (2006). Regulation of seed size by hypomethylation of maternal and paternal genomes. Plant Physiol..

[97] Xiao W., Custard K.D., Brown R.C., Lemmon B.E., Harada J.J., Goldberg R.B., Fischer R.L. (2006). DNA methylation is critical for *Arabidopsis* embryogenesis and seed viability. Plant Cell.

[98] Gehring M., Bubb K.L., Henikoff S. (2009). Extensive demethylation of repetitive elements during seed development underlines gene imprinting. Science.

[99] Ogneva Z.V.S., Dubrovina A.S., Kiselev K.V. (2016). Age-associated alterations in DNA methylation and expression of methyltransferase and demethylase genes in Arabidopsis thaliana. Biol. Plant..

[100] Varghese B., Naidoo C., Pammenter N.W. (2016). The use of plant stress biomarkers in assessing the effects of desiccation in zygotic embryos from recalcitrant seeds: Challenges and considerations. Plant Biol..

[101] Leprince O., Buitink J., Hoekstra F.A. (1999). Axes and cotyledons of recalcitrant seeds of *Castanea sativa* Mill. exhibit contrasting responses of respiration to drying in relation to desiccation sensitivity. J. Exp. Bot..

[102] Dou L., Jia X., Wei H., Fan S., Wang H., Guo Y., Duan S., Pang C., Yu S. (2017). Global analysis of DNA methylation in young (J1) and senescent (J2) *Gossypium hirsutum* L. cotyledons by MeDIP-Seq. PLoS ONE.

[103] Obroucheva N., Sinkevich I., Lityagina S. (2016). Physiological aspects of seed recalcitrance: A case study on the tree *Aesculus hippocastanum*. Tree Physiol..

[104] El-Maarouf-Bouteau H., Mazuy C., Corbineau F., Bailly C. (2011). DNA alteration and programmed cell death during ageing of sunflower seed. J. Exp. Bot..

[105] Madugundu G.S., Jean Cadet J., Wagner J.R. (2014). Hydroxyl-radical-induced oxidation of 5-methylcytosine in isolated and cellular DNA. Nucleic Acids Res..

[106] Erdmann R.M., Souza A.L., Clish C.B., Gehring M. (2015). 5-Hydroxymethylcytosine is not present in appreciable quantities in *Arabidopsis* DNA. Genes Genomes Genet..

[107] Walters C.H., Berjak P., Pammenter N., Kennedy K., Raven P. (2013). Preservation of recalcitrant seeds. Science.

[108] Corbineau F. (2002). Markers of seed quality: From present to future. Seed Sci. Res..

[109] Birti S., Colville L., Pritchard H.W., Pearce S.R., Kranner I. (2011). Mathematically combined half-cell reduction potentials of the low-molecular-weight thiols as markers of seed aging. Free Radic. Res..

